# Ionic Substitutions in Non-Apatitic Calcium Phosphates

**DOI:** 10.3390/ijms18122542

**Published:** 2017-11-27

**Authors:** Aleksandra Laskus, Joanna Kolmas

**Affiliations:** Department of Inorganic and Analytical Chemistry, Faculty of Pharmacy with Laboratory Medicine Division, Medical University of Warsaw, ul. Banacha 1, 02-097 Warsaw, Poland; aleksandralaskus@gmail.com

**Keywords:** calcium phosphates, ionic substitution, brushite, αTCP, βTCP, bioceramics

## Abstract

Calcium phosphate materials (CaPs) are similar to inorganic part of human mineralized tissues (i.e., bone, enamel, and dentin). Owing to their high biocompatibility, CaPs, mainly hydroxyapatite (HA), have been investigated for their use in various medical applications. One of the most widely used ways to improve the biological and physicochemical properties of HA is ionic substitution with trace ions. Recent developments in bioceramics have already demonstrated that introducing foreign ions is also possible in other CaPs, such as tricalcium phosphates (amorphous as well as α and β crystalline forms) and brushite. The purpose of this paper is to review recent achievements in the field of non-apatitic CaPs substituted with various ions. Particular attention will be focused on tricalcium phosphates (TCP) and “additives” such as magnesium, zinc, strontium, and silicate ions, all of which have been widely investigated thanks to their important biological role. This review also highlights some of the potential biomedical applications of non-apatitic substituted CaPs.

## 1. Introduction

Calcium phosphates (CaPs) are commonly used biomaterials in various medical fields, i.e., mineralized tissue surgery, implantology, orthopaedics, and stomatology. Due to their special properties, such as biocompatibility, bioactivity, nontoxicity, and osteoconductivity, they play a crucial role as bone grafts, bone fillers, and coating materials [1,2]. Most commonly, they are applied in a form of porous granules, scaffolds, or hydraulic, ready-to-use, mouldable cements. What is more, they may serve as local drug delivery systems to introduce medicines directly into the mineralized tissue [3,4,5].

**Scheme 1:**
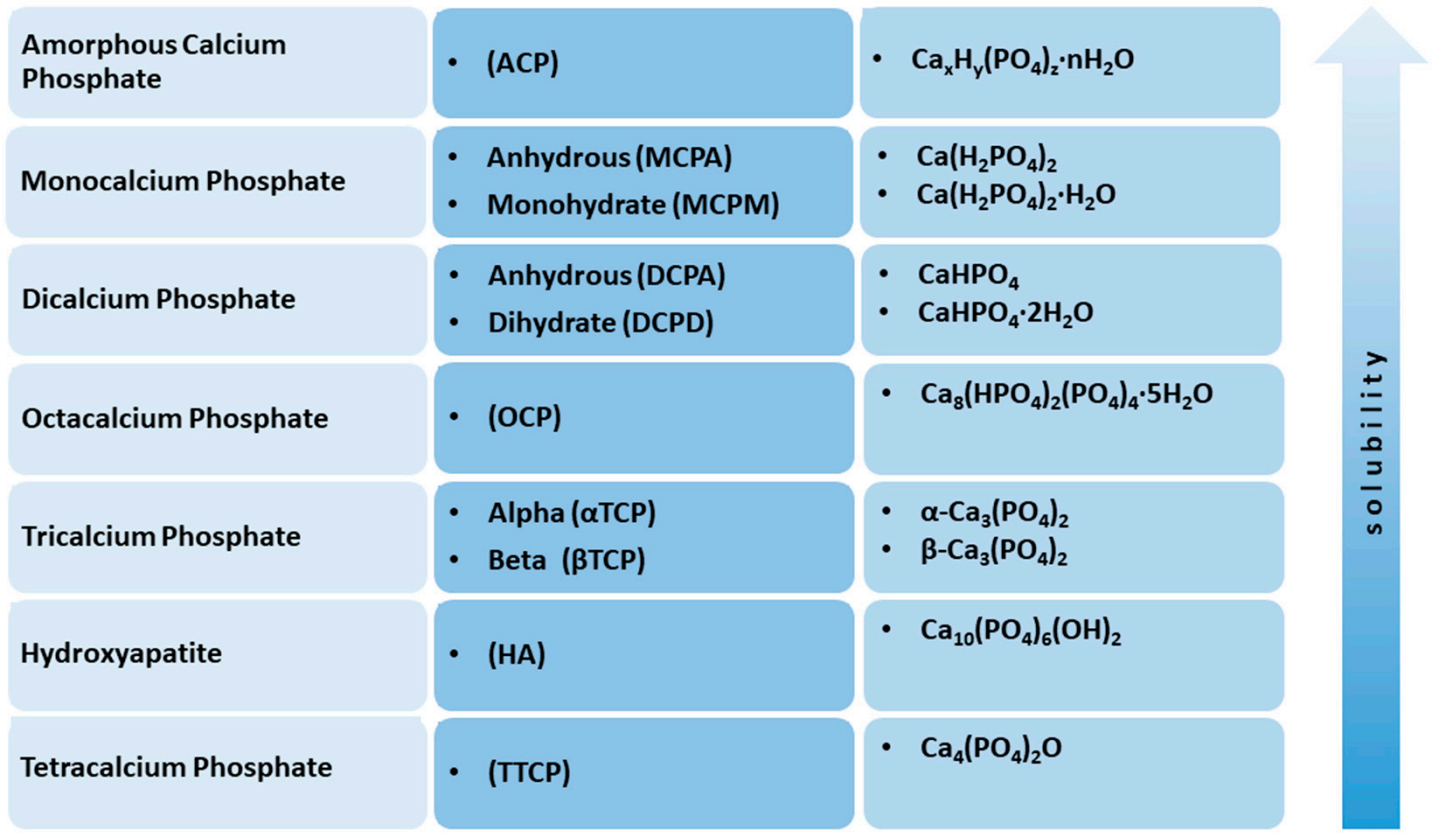
Calcium phosphates of biomedical interest.

CaPs can be obtained in different crystalline or amorphous phases, depending on the synthesis conditions (see Scheme 1). These materials differ in Ca/P molar ratio and both physicochemical and biological properties, such as solubility, biodegradability, and bioactivity [1]. Moreover, they are stable in various pHs: for example, HA is chemically stable in aqueous solution in pH > 8, whereas octacalcium phosphate (OCP) and dicalcium phosphate dihydrate (DCPD) in neutral and pH < 6, respectively. HA and tetracalcium phosphate (TTCP) are almost insoluble CaPs (solubility at 25 °C is for HA and TTCP 0.0003 and 0.0007 g/dm^3^). Regarding the easily soluble materials, amorphous calcium phosphate, monocalcium phosphate monohydrate and anhydrous (ACP, MCPM, and MCPA, respectively) should be mentioned [1].

Among all CaPs, hydroxyapatite (HA) with the formula Ca_10_(PO_4_)_6_(OH)_2_ is the most thoroughly studied material. Thanks to its similarity to the biological apatite, the main constituent of the inorganic part of human mineralized tissues, HA is an attractive material used alone as a bioceramic or as a component of hybrid composites for biomedical engineering [6,7,8]. It is thoroughly documented that the chemical and biological properties of HA may be improved by the incorporation of foreign ions into its crystal structure [9,10]. In order to provide a few examples, the introduction of Mn^2+^ favours the activity and proliferation of osteoblasts and has a positive impact on the osteointegration process [11]. Fe ions improve the response of osteoblasts [9,11]. In turn, doping CaPs with selenium may give the material an anticancer potential [9,12]. Moreover, the slight replacement of Ca^2+^ with Ag^+^ cations in the HA structure results in gaining additional antibacterial properties by the material [9,13].

Recently, it turned out that other calcium phosphates, i.e., tricalcium phosphates (TCPs) and dicalcium phosphate dihydrate (DCPD), also possess a natural ability to exchange ions within their crystal lattice. Due to the fact that these CaPs are more soluble than HA [1], it gives an opportunity to create a more resorbable material, releasing therapeutic agents with favourable kinetics.

Several reviews with different approaches have been published on the topic of substituted calcium phosphates. Some of them focused on various ionic substitution in HA [9], antibacterial ions introduced into the HA crystal lattice [14], biological activity of substituted HA [15], or ion-substituted calcium phosphate coatings [16].

In turn, the aim of this work is to review the attempts made so far in the field of ionic substitutions in non-apatitic CaPs and to summarize their effects. To begin with, the biological role of the most relevant ionic substitution, followed by a short presentation of the main, non-apatitic CaPs used in biomedical applications will be presented. The final section will report the recent achievements in ionic modification of non-apatitic CaPs.

## 2. The Most Relevant Ionic Substitution within the CaPs Crystal Structure

Comprehensive studies on the composition of bone and dental tissues have led to the conclusion that biological apatite is not a pure, stoichiometric HA, but is instead, substituted by different ions, including CO_3_^2−^, HPO_4_^2−^, SiO_4_^2−^, Mg^2+^, K^+^, Na^+^, Zn^2+^, Mn^2+^, F^−^, or Cl^−^ [1,17]. The quantitative and qualitative content of these “impurities” varies according to the age and condition of the tissue. However, it should be concluded that all of them play a crucial role in mineralized tissue metabolism [17]. Therefore, recent developments in ionic substitutions in synthetic CaP materials have mainly considered these ions that naturally occur in biological apatites. In the following sections, the most ubiquitously introduced ions will be presented.

### 2.1. Magnesium (Mg^2+^)

Magnesium is the fourth most abundant element in the human body. Significant amount of this element is present in bone, dentin, and enamel. As much as 60% of the body’s total Mg is deposited in bone, which acts as a natural reservoir for the metal. Skeletal magnesium is located either on the surface of hydroxyapatite or in the hydrated layer around the crystal [13,18,19]. Magnesium contributes to the maintenance of homeostasis in mineralized tissues. An appropriate level of the metal is vital for preventing osteoporosis and other bone tissue impairments. The element’s influence on mineralized tissue is both direct and indirect [19]. When it comes to the direct influence, magnesium has a significant impact on bone crystal structure and skeletal cell activity. Magnesium deficiency leads immediately to hypomagnesaemia, which is compensated through the mobilization of bone deposits [20]. This, in turn, leads to the alteration of the biological apatite structure and, as a result, a decrease in mechanical strength. Both in vitro and in vivo studies have confirmed the element’s influence on bone cell activity. Magnesium stimulates osteoblast activity [21]. On the other hand, metal deficiency promotes low-grade inflammation and, as a consequence, an increase in osteoclast activity, which contributes to bone mass loss [19,20,21]. Among its indirect effects, magnesium has an impact on the secretion of parathyroid hormone (PTH) and thereby a secretion of 1.25(OH)_2_ vitamin D. Experimental evidence show that the metal shortages lower the concentration of both PTH and vitamin D in serum, thereby leading to hypocalcaemia [19,20].

### 2.2. Zinc (Zn^2+^)

Zinc is the second most abundant trace metal in the human body. Approximately 86% of the total amount of this element is localized in skeletal muscles and bone tissue. The highest proportion of Zn^2+^ ions within the bones is localized in the osteoid and non-mineralized matrix [13,22]. Zinc acts as a cofactor for numerous enzymes and, due to this, is involved in DNA and RNA replication, protein synthesis, and bone metabolism. The element promotes both differentiation and proliferation of osteoblasts [2]. The metal’s stimulating effect on osteoblastic differentiation has been partially attributed to its ability to augment the expression of runt-related transcription factor 2, which is a key transcription factor in osteoblastogenesis. In vitro studies showed that zinc also stimulates the osteoblastic production of growth factors, i.e., insulin-like growth factor (IGF-I) and transforming growth factor (TGF-β), both of which promote bone formation [22,23]. Experimental evidence show that zinc exhibits a stimulating effect on alkaline phosphatase (ALP), which promotes bone mineralization through the dephosphorylation of organic pyrophosphates. Simultaneously, Zn^2+^ ions suppress osteoclastic activity. In vitro studies revealed that zinc inhibits osteoclastic resorption mediated by PTH and proinflammatory cytokines [22]. Studies in vivo confirmed zinc’s specific impact on bone cell activity and its contribution to the normal development of the skeletal system. Experiments conducted with chicks [24] and rats [25] showed that Zn supplementation increases the strength parameters of long bones, decrease extracellular bone resorption markers, and augments the level of bone formation indicators.

### 2.3. Strontium (Sr^2+^)

Strontium is a trace element present in significant amounts in calcified tissues [13,26]. This particularly concerns the mineral phase of bone, which is characterized by high metabolic turnover. A new developing bone is likely to contain higher concentrations of Sr than a mature one [26]. Low doses of strontium contribute to proper bone formation, while higher amounts are believed to cause osteomalacia [26,27]. It is noteworthy that strontium ranelate is one of the drugs administered to treat postmenopausal osteoporosis. It reduces significantly the risk of vertebral and non-vertebral fractures. Strontium ranelate exhibits a specific, dualistic influence on mineralized tissues. On the one hand, it stimulates bone formation, while on the other, it decreases its resorption. Studies in vitro showed that this particular dual-mode action of the element is caused by its stimulating effect on osteoblastic differentiation and simultaneous inhibition of osteoclastic activity [28]. What is more, experiments conducted on osteoarthritic, subchondral bone osteoblasts revealed that strontium ranelate decreases the expression of key factors affecting bone resorption [29]. An interesting in vivo study with a zebrafish as an animal model was also designed. The outcomes of the experiment demonstrated an increase in vertebral mineralization when compared with the control with lower strontium concentrations. Higher doses of strontium caused inhibited mineral deposition in dose-dependent order [30]. All of the facts mentioned above make strontium ranelate one of the drugs that is still administered as a treatment of osteoporosis [31].

### 2.4. Silicon (SiO_3_^2−^, SiO_4_^2−^)

Silicon is the third most abundant trace element in the human body [32]. It significantly affects bone metabolism and contributes to proper mineralized tissue formation. A particularly high concentration of silicon can be found in metabolically active skeletal cells. A relatively high level of the element is found in the mitochondria of these cells. A pioneer in investigating silicon’s influence on bone metabolism was Carlisle [33,34,35,36]. In vitro and in vivo studies conducted by Carlisle [33,34,35,36] proved that silicon is localized in active bone growth areas. This, in turn, suggested that Si plays a physiological role in the mineralization process. What is more, the scientists observed that silicon deficiency contributes to problems with normal growth and skeletal development abnormalities [32,33,37]. Over the years, Carlisle’s observations have been confirmed. Dietary silicon can be absorbed in the form of orthosilicic acid (H_4_SiO_4_). Studies [32,37,38] have investigated the influence of this acid on bone metabolism. Experiments conducted in osteoblastic cell line MG-63 showed that orthosilicic acid promotes the synthesis of collagen type 1 and augments osteoblastic differentiation markers, i.e., alkaline phosphatase and osteocalcin levels [37]. Silicon does not affect the expression of the collagen type 1 gene, but it does stimulate the prolyl hydrolase involved in collagen synthesis [32]. In vivo studies also confirmed a stimulating effect of the element on collagen type 1, including randomized trials conducted in osteopenic women [38]. Overall, an adequate Si level is crucial for maintaining higher bone mineral density (BMD). It contributes to bone mineralization and accelerates the calcification rate [39,40,41,42].

## 3. Non-Apatitic Calcium Phosphates

Unlike biological apatites, non-apatitic calcium phosphates are not present in normal mineralized tissues. They frequently occur in pathological calcifications such as dental calculi, urinary stones or heart valve calcifications [43]. Non-apatitic CaPs, except crystalline TCP and TTCP, can be easily synthesized using standard wet methods, by adjusting the Ca/P ratio, the pH of the solution and the temperature beforehand [44]. As was mentioned above, the various types of non-apatitic CaPs differ in their solubility, which decreases as follows: ACP < MCPM < MCPA < DCPD < OCP < αTCP < TTCP < βTCP [45]. In general, non-apatitic CaPs are more soluble and less stable than hydroxyapatite, which allows them transform easily into apatites and substituted apatites or other calcium phosphates under physiological conditions through the dissolution-precipitation process [44].

### 3.1. Amorphous Calcium Phosphate (ACP)

Amorphous calcium phosphate (ACP) is a hydrated, thermodynamically unstable, transient phase that commonly precipitates during the formation of more stable CaPs in aqueous systems. The structure of ACP is still being discussed in the literature [46]. Briefly, it is proposed that the main structural unit has a nearly spherical cluster measuring 9.5 Å in diameter with the basic composition of Ca_9_(PO_4_)_6_ [47]. Moreover, the clusters are interspersed with water molecules (in various contents) [48]. The presence of acidic phosphates in the ACP structure is also under investigation. ACP can be obtained by wet precipitation in aqueous medium at low temperature (the wet method) and by using high energy processing and high temperature (the dry method) [48,49]. As the wet method is more ubiquitously applied, only this route will be briefly outlined below. Wet synthesis may be undertaken either in aqueous medium or a water-alcohol solution. Usually, it consists of two steps: rapid mixing of the reagents and precipitate filtration. Low or room temperature, high supersaturation, and a pH close to 10 are required. The reaction consists of a double decomposition of calcium (e.g., Ca(NO_3_)_2_·4H_2_O) and phosphate (e.g., (NH_4_)_2_HPO_4_) salt in the aqueous or water-alcohol medium [48,49]. After rapid mixing and precipitate filtration, the powder is lyophilized. The prepared ACP is stored in a freezer in order to prevent conversion or phase transformation. The ACP’s structure and chemical composition depends on the pH and composition of the mother solution. Hence, the Ca/P ratio of the synthesized ACP can range from 1 to 2, or even higher. Having said this, the most commonly known Ca/P ratios are 1.5 and 1.33 [48]. The Pβ diffractogram of ACP is typical for amorphous materials: it only presents a very broad line with no narrow reflections [47].

Due to the significant chemical and structural similarities to calcified tissues, as well as its excellent biocompatibility and bioresorbability, ACP is commonly used as a component of calcium phosphates cements (CPCs) in surgery or dentistry. Owing to the fact that it can easily transform into biological apatites, it could also be a promising material for the artificial bone grafts engineering. According to the literature, several mechanisms of ACP transformation into crystalline apatite are proposed, depending on pH or the presence of other ions [47,48]. In several cases, the intermediate phase is OCP [48]. It should be also noted, that ACP occurs naturally in soft tissue pathological calcifications, i.e., heart valve calcifications [43,49,50].

### 3.2. Dicalcium Phosphate Dihydrate (DCPD)

**Figure 1:**
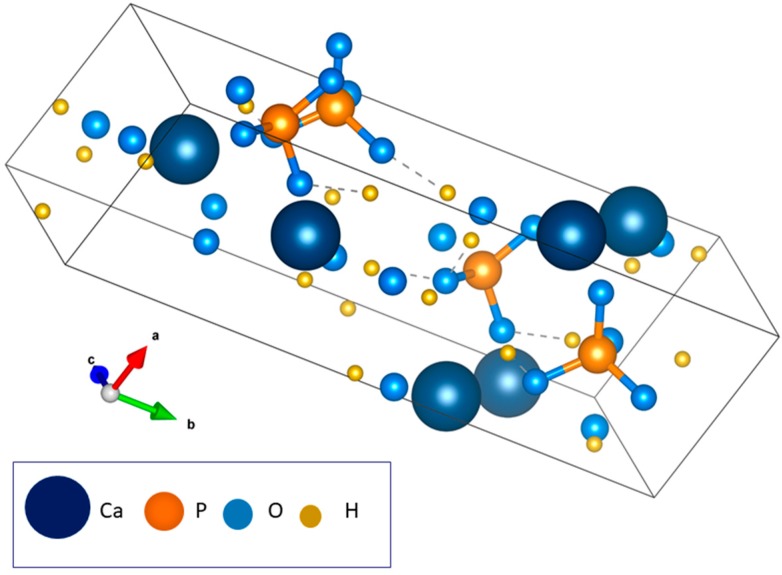
Crystalline structure of dicalcium phosphate dihydrate (DCPD).

Dicalcium phosphate dihydrate, also known as brushite or calcium hydrogen phosphate dihydrate, is a crystalline calcium phosphate that can be described using the formula CaHPO_4_·2H_2_O. It crystallizes in the monoclinic *Ia* space group (see Figure 1). The DCPD crystals consist of CaP chains arranged parallel to each other, with water molecules situated between the chains [47].

DCPD is naturally present in dental calculi, urinary stones and precipitations involved in chondrocalcinosis [43]. In laboratory terms, it crystallizes easily from aqueous solutions and is stable in acidic environments, namely the pH between 4 and 6. Most commonly, it is obtained by mixing calcium chloride CaCl_2_ with NaH_2_PO_4_ or NH_4_H_2_PO_4_ in a water solution. The synthesis is carried out according to the following chemical reaction [51]:

Na_2_HPO_4_ + CaCl_2_ + 2H_2_O → CaHPO_4_·2H_2_O + 2NaCl

Apart from the starting materials listed above, other calcium and phosphate sources can also be employed. Among them, calcium hydroxide (Ca(OH)_2_), calcium nitrate (Ca(NO)_3_) or calcium acetate (CH_3_COOH)_2_Ca and phosphoric acid (H_3_PO_4_) should be mentioned [49,52,53,54]. As demonstrated above, the reagents are mixed in an equimolar ratio. The proper pH value is usually maintained by the addition of HNO_3_, HCl, H_3_PO_4_, and KOH or NH_4_OH. The literature provides data showing that brushite forms at the pH of 5 and the temperature of 37 °C. In order to synthesize DCPD at the pH of 6, the temperature should be lowered to 25 °C [55].

Brushite can be also obtained by mixing two different calcium phosphate powders in water [54]. The starting materials are βTCP and monocalcium phosphate hydrate (Ca(H_2_PO_4_)_2_·H_2_O). Sodium pyrophosphate (Na_2_H_2_PO_4_) is added to the mixture as a setting regulator. The synthesis may be conducted in a sulphuric acid solution or in inorganic (silica) and organic (collagen) gels [55].

It should be noted that brushite is a metastable material. It transforms easily into the anhydrous form monetite (CaHPO_4_). Dehydration begins already in atmospheric conditions [51]. Under the physiological pH ranging from 7 to 7.5, DCPD transforms into HA. An essential aspect is that it resorbs much faster than apatite and, due to this, is widely applied in surgery as a CPC component. Moreover, in dentistry it is used as an anti-plaque factor in toothpastes [43,55,56].

### 3.3. Tricalcium Phosphate (TCP)

**Figure 2:**
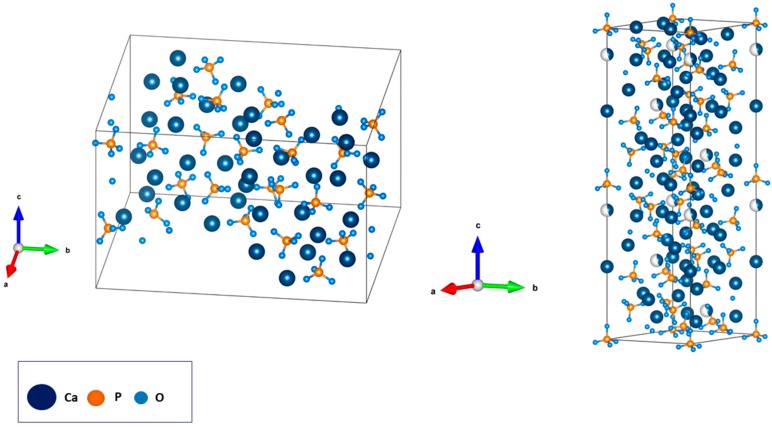
The crystalline structures of tricalcium phosphate (TCP): α form (**left**) and β form (**right**).

Tricalcium phosphate, which can be described with the general formula Ca_3_(PO_4_)_2_, exists in two allotropic forms, i.e., α and βTCP, both of which are of great importance from the biological point of view. Schematic models of the crystal structures of these materials are presented in Figure 2. αTCP crystallizes in the monoclinic space group P2_1_/a, whereas βTCP possesses a rhombohedral structure [47].

As the hydroxyapatite earned its immense popularity in biomedicine due to its great similarity to biological apatite, TCP is still gaining interest thanks to its relatively high solubility. Both HA and TCP form a strong bond with the mineralized tissue and favour bone formation. Due to the poor resorbability of HA, both CaPs are often mixed together in different ratios in order to increase the bioreactivity of the material [57,58,59].

αTCP is a high temperature allotropic form, which comes into being during the heat treatment of βTCP above 1125 °C [60]. The synthesis of αCa_3_(PO_4_)_2_ brings one major problem, which is the metastability of the obtained material under the room temperature. Its thermal stability ranges from 1430 to 1470 °C. Thus, synthesizing αTCP at a high temperature requires immediate cooling to stabilize the structure after the previous heat treatment [60,61]. αTCP is highly soluble and together with a liquid phase forms a hard and stable material that is used in bone cements. It should be also noted that αTCP transforms into CDHA in water medium according the following reaction [60]:

3Ca_3_(PO_4_)_2_ + H_2_O → Ca_9_(PO_4_)_5_(HPO_4_)OH

αTCP can be obtained via different methods, i.e., precursor’s thermal transformation, solid-state precursors’ reaction and self-propagating, high-temperature or combustion synthesis [61]. When it comes to thermal transformation, the decomposition may consider CDHA, ACP or βTCP (see Equations (1)–(3)):

Ca_9_(HPO_4_)(PO_4_)_5_(OH)_(s)_ → 3αCa_3_(PO_4_)_2(s)_ + H_2_O_(g)_ (T ≥ 1150 °C) (1)

Ca_9_(PO_4_)_6_·nH_2_O_(s)_ → αCa_3_(PO_4_)_(s)_ + nH_2_O_(g)_ (600 °C ≤ T ≤ 800 °C) (2)

βCa_3_(PO_4_)_(s)_ → αCa_3_(PO_4_)_(s)_ (1125 °C ≤ T ≤ 1430 °C (3)

Both the decomposition of CDHA (Equation (1)) and ACP (Equation (2)) are commonly applied [57]. Starting materials are usually synthesized using the wet precipitation method. Nonetheless, high temperature, βTCP to αTCP crystalline phase transition is the most direct and the simplest way to obtain αCa_3_(PO_4_) [62,63,64,65,66]. 

Considering obtaining αTCP via solid-state reaction, the most commonly applied synthesis routes are listed below [67,68,69,70,71].

CaCO_3(s)_ + 2CaHPO_4(s)_ → αCa_3_(PO_4_)_2(s)_ + CO_2(g)_ + H_2_O_(g)_

3CaCO_3(s)_ + 2NH_4_H_2_PO_4(s)_ → αCa_3_(PO_4_)_2(s)_ + 3CO_2(g)_ + 3H_2_O_(g)_+ 2NH_3(g)_

CaCO_3(s)_ + Ca_2_P_2_O_7(s)_ → αCa_3_(PO_4_)_2(s)_ + CO_2(g)_

Ca_10_(PO_4_)_6_(OH)_2(s)_ + 2CaHPO_4(s)_ → 4αCa_3_(PO_4_)_2(s)_ + 2H_2_O_(g)_

In general, solid-state reaction scheme consists of a few stable steps: milling the powder mixture, putting the ground mixture under pressure and finally heating the powder above the transformation temperature. The first two stages serve to reduce the particles size, promote proper homogenization and increase the contact area. When it comes to the heat treatment parameters, the recommended temperature ranges from 1250 to 1500 °C. In turn, the sintering time may vary from 2 to 48 h. To avoid the reversion of the crystal phase, quenching should be employed immediately [61].

Less common ways of obtaining αTCP are self-propagating, high-temperature, and combustion synthesis [72,73,74,75]. Briefly, they consist in preparing a mixture of Ca and P sources in a proper ratio, either as a solid pellet or solution, and the heat treatment. In case of the self-propagating, high temperature method, the heat is applied from an external source, while in case of the self-combustion route, the reagent solution contains an organic, inflammable fuel (e.g., urea) that acts as a catalyst for the self-combustion of the mixture [72,73,74,75].

Recently, some novel approaches to βTCP synthesis have been made [51,76,77,78]. Herein, they will be briefly presented, together with more conventional and ubiquitously employed methods. In general, βTCP synthesis methods include:

- Thermal transformation of the precursor;
- Solid-state synthesis;
- Wet chemical precipitation;
- Sol-gel technique;
- Self-combustion method.

The most conventional methods from the above are thermal transformation of the precursor, solid-state synthesis and wet chemical precipitation. Most frequently, thermal transformation of the precursor consists in calcination of previously precipitated CDHA at the temperature above 800 °C [55,77]. When it comes to solid-state synthesis, possible reactions schemes that can be employed are similar to those presented for αTCP. The only differences concern the heat treatment parameters, which should be maintained at the level preventing βTCP to αTCP phase transition. In turn, wet chemical precipitation consists in precipitating CaP at a Ca/P molar ratio of ≈1.5 followed by the product’s calcination. 

Another interesting method is a sol-gel technique [77]. In this case, the precipitation is carried out in very acidic conditions. The proper pH value is usually achieved by the addition of citric acid or concentrated nitric acid, and varies from 2 to 3. The sol-gel transformation is caused by vigorous mixing of Ca and P sources in a liquid medium during the simultaneous heating up to 80 to 90 °C [76,77].

The self-combustion method has already been described. An alternative to this method is microwave self-combustion synthesis [78].

An essential property of TCP is that, similarly to hydroxyapatite, it forms a strong bond with mineralized tissue and favours bone formation. Due to its relatively high solubility, it is commonly applied as a component of calcium phosphate cements and other bone substitutes [56,57,61]. 

## 4. Ionic Substitutions in Non-Apatitic Calcium Phosphates

### 4.1. Substituted Amorphous Calcium Phosphate (ACP)

As mentioned above, unsubstituted ACP cannot be formed at the physiological pH level. It usually forms at the alkaline pH of about 10. Introducing some specific ions, i.e., Mg^2+^, Sn^2+^, Al^3+^, P_2_O_7_^4−^, and CO_3_^2−^, stabilizes the ACP and inhibits its transformation into HA [48]. Sometimes, such a modification can also promote hydrolysis to brushite instead of apatite. Lee and Kumta [51] decided to modify ACP with Mg^2+^ ions mainly because of their excellent biocompatibility. They used a precipitation method, with MgCl_2_ as a source of magnesium. The concentrations of Ca and P in the two conducted experiments corresponded to the composition of HA (Ca/P = 1.67) and TCP (Ca/P = 1.5). The magnesium content was maintained at the level of 30 mol % and 20 mol % for the HA and TCP, respectively. Physicochemical analysis was carried out. The results revealed that Mg ions act as a phase stabilizer. During the heat treatment of ACP, αTCP is known to form at 600 °C. The addition of Mg^2+^ stabilized MgTCP at this temperature and retarded its transformation [51]. 

The in vitro biological activity of Mg-substituted ACP, amorphous magnesium phosphate (AMP) and pure HA was tested on MC3T3-E1 preosteoblasts [79]. In general, the results for amorphous materials (Mg-substituted ACP and AMP) have demonstrated higher proliferation and differentiation rate as well as higher mineralization of preosteoblast cells than HA samples. 

The effect of silicon doping on the transformation of ACP to αTCP has been investigated by Dong et al. [80]. During the synthesis of HA with high silicon concentration, the amorphous phase enriched with Si was easily formed and then transformed to αSiTCP at lower temperatures than SiHA.

Kato et al. [81] have prepared potassium-substituted ACP for potential application in dentin hypersensitivity treatment. It has been shown that potassium release from amorphous material is significantly larger than from potassium-substituted hydroxyapatite. ACP enriched in silver ions was prepared via chemical precipitation method by Yu et al. [82]. The obtained AgACP material, together with slightly acidic compounds were then used to produce calcium phosphate cements (CPCs). The sufficient silver release and high cytotoxic effect toward *Escherichia coli* were demonstrated only in the samples prepared without heat treatment.

### 4.2. Substituted Dicalcium Phosphate Dihydrate (DCPD)

Modifying brushite with foreign ions is also an object of scientific interest. DCPD is a material commonly used as a component of CPCs, as it transforms easily to HA under physiological conditions. The most ubiquitously applied ionic substitution in case of brushite is magnesium, mainly due to the potential enhancement of the bioactivity of such a modified material [51,83,84,85,86]*.* Some studies also deal with the introduction of silicon [87], strontium [88,89], cuprum [90], zinc [91], and iron [92]. Recently, a study concerning the introduction of nickel has been also conducted [93]. Alkhraisat and Cabrejos-Azama focused on magnesium-modified, brushite-based CPC [83,84,85]. Various ways of introducing Mg^2+^ ions into brushite cements were investigated and reported, also. 

Alkhraisat et al. [83] used TCP and magnesium-substituted TCP as substrates in this process. In their studies, the preparation of brushite cement consisted of the following few steps:

- Preparation of βTCP and βMgTCP powder via solid-state synthesis;
- Addition of MCPM to previously crushed and mixed powders;
- Addition of water to the composed powder;
- Mixing in mortar.

By modifying the substrate (TCP) with different amounts of Mg^2+^, Alkhraisat et al. [83] managed to replace 3% of the Ca^2+^ with Mg^2+^ ions in the final product. The final setting time of the CPC increased, together with its magnesium content. Moreover, the release rate of the ions was affected. The release of Ca^2+^ decreased as the Mg^2+^ concentration increased, which is a result of magnesium’s inhibiting influence on the dissolution of brushite. A similar observation was made by Lee and Kumta in their work [51]. Hence, it could be stated that Mg^2+^ acts as a phase stabilizer of DCPD.

In turn, Correia et al. [94] studied the role of Mg^2+^ ions on the growth of acidic calcium phosphate crystals: OCP and DCPD. It was shown that the presence of Mg^2+^ could affect the morphology of the coatings obtained by electrodeposition. Magnesium inhibited the OCP and DCPD crystal growth due to surface adsorption and doping process, respectively [94].

**Figure 3:**
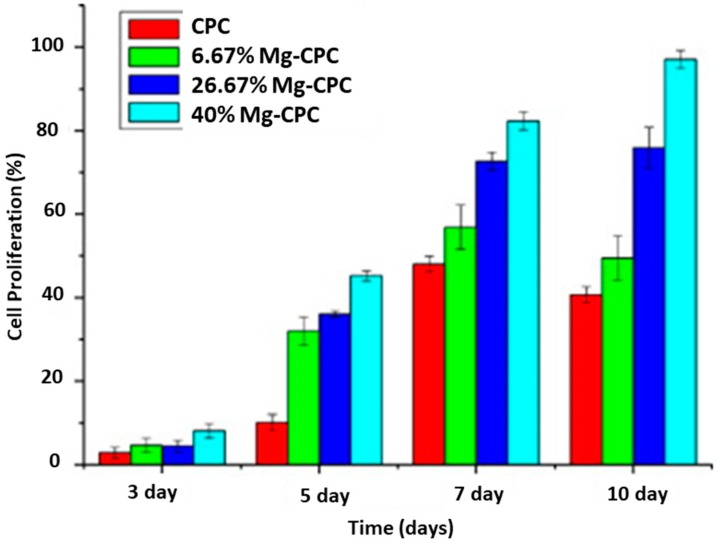
Cell proliferation after seeding on calcium phosphates cement (CPC) and MgCPCs (error bars represent SD). Reprinted from [84] with permission from Elsevier.

**Figure 4:**
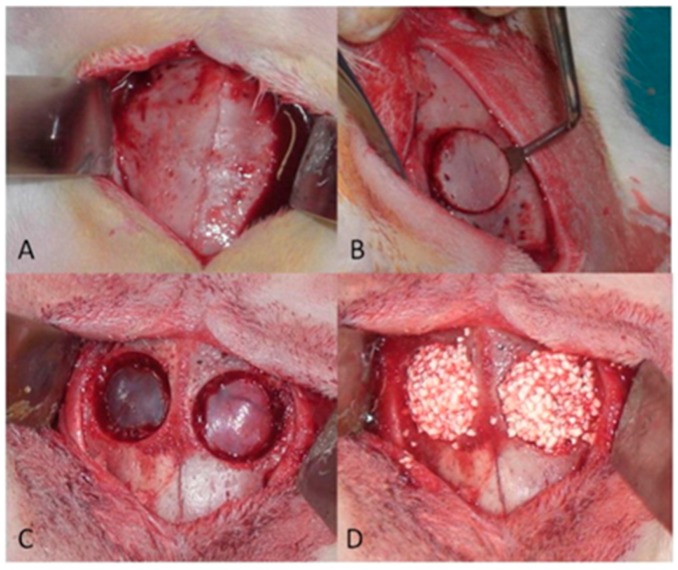
(**A**) Rabbit calvaria exposed; (**B**) removal of bone block; (**C**) bone defects exposed; (**D**) bone defects filled with bone substitute. Reprinted from [84] with permission from Elsevier.

In the study [84], the biological properties of magnesium-modified brushite cement were examined (see Figure 3 and Figure 4). The investigation involved cell studies and animal model experiments. The evaluation of the in vitro response to the cement was conducted using the osteoblastic cell line MG-63. In turn, in vivo tests were performed using a rabbit model. Both in vitro and in vivo studies proved that there was an increased biological response in case of MgCPC in comparison with unmodified CPC. Specifically, cell proliferation, cell adhesion, and bone formation all increased. No manifestation of inflammation was observed [84].

Alkhraisat et al. [88] also carried out studies concerning the impact of strontium and pyrophosphates on the physicochemical properties of brushite cement. The results showed that both Sr^2+^ and P_2_O_7_^2−^ inhibit the cement setting reaction.

**Figure 5:**
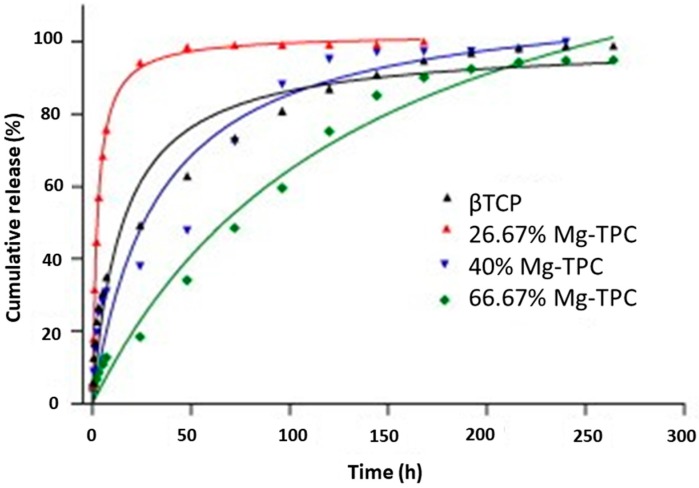
Release of vancomycin from different CPCs. Reprinted from [85] with permission from Elsevier.

Cabrejos-Azama et al. [85] used magnesium-substituted DCPD cement as a drug carrier. Vancomycin, an antibiotic commonly used against *Staphylococcus aureus*, which is one of the most frequent pathogens associated with mineralized tissue infections, was chosen as a model drug. The cement was loaded with the antibiotic through the adsorption from the solution or introduction into the solid phase of cement. The release profile varied together with Mg content in the TCP used as one of the reagents in the preparation of brushite cement (see Figure 5). The material prepared with βMgTCP containing 66.67% Mg^2+^ released vancomycin with zero-order kinetics. Vancomycin was also used as a drug model in study [87], in which silicon was chosen as an ionic modifier. The investigated material was CPC-silica gel composite. The composite was obtained through the infiltration of the macropores of the CPC by the silica gel. The infiltration process altered both density of the material and the release mode of vancomycin. In comparison with unmodified CPC, 25% of previously introduced drug remained in the SiCPC matrix. The cytocompatibility of obtained composite was also examined. The composites with the highest silicate content performed as high cell proliferation as hydroxyapatite, which was one of the reference materials. However, biological response did not correlate with the release rate of silicon from the material, but seemed to be attributed to the phosphate release and magnesium absorption from the cell culture medium [87].

The available literature contains also the co-substituted DCPD cements information [95,96]. Torres et al. [95] focused on co-doping DCPD with the combination of Mn^2+^ and Sr^2+^. The injectable cement contained also an addition of sucrose, which improved its biological and mechanical properties. In turn, Vahabzadeh et al. [96] studied the influence of Si and Zn ions as dopants of DCPD on the physical, biological, and mechanical properties of cements. In vivo results revealed that Si and Zn addition may improve the early stage osseointegration process [96].

### 4.3. TCP Substituted with Foreign Ions

Ionic substitution in both α and βTCP is currently being investigated. A solid-state reaction, as well as the heat treatment of already precipitated powders, are commonly applied to synthesize these materials [97,98,99,100,101,102,103,104,105,106,107,108,109,110,111,112,113,114,115,116,117,118,119,120,121,122,123,124,125,126,127,128,129,130,131,132,133,134,135,136,137,138,139,140,141,142,143,144,145,146]. It is worth underlining that there are two main directions in ionic modification of α and βTCP. One of them is improving the properties of CaP, while the second one is stabilizing the phase. In the following paragraph the most interesting studies on this topic will be briefly discussed.

#### 4.3.1. αTCP Substituted with Different Ions

Various elements, including Mg, Sr, Si, and Ag, are being successfully introduced into αTCP’s crystal lattice. It should be underlined that Si is the most widely applied and thoroughly examined dopant. 

αSiTCP samples can be obtained using various wet and solid-state methods [98,99,100,101,102,103]. Reid et al. [98] prepared Si-substituted αTCP using one of the most ubiquitous ways of synthesis, i.e., by sintering previously precipitated precursor, which was Si-enriched CaP. The applied temperature was 1250 °C, and the single-phase samples contained 0.59–1.14 wt % of Si. 

As stated before, pure αTCP is stable only in the range of 1430 to 1470 °C. Thus, it is important to note that the addition of silicon favours the formation of αTCP and helps to stabilize its structure in lower temperature [97]. The chemical reactivity of αSiTCP was also examined by Moitsuke et al. [100]. αTCP is known to convert easily to calcium-deficient hydroxyapatite in water solution. However, the study showed that, in comparison with pure αTCP, the reactivity of αSiTCP was significantly lower. This could be related to the release of silicon and formation of an amorphous Si layer slowing down the dissolution-precipitation reaction. 

Interesting conclusions were made by Reid et al. in their work [105]. The study considered the influence of the presence of magnesium impurities in one of the reactants on the phase composition of obtained powder. To synthesize the final material, the sol-gel technique combined with the appropriate heat treatment was applied. Surprisingly, it turned out that an Mg content of barely 250 to the chemical structure of αSiTCP was investigated by Duncan et al. [99], who obtained high-purity αSiTCP through a solid-state reaction in a furnace at 1300 °C. Detailed physicochemical analysis (X-ray diffraction with Rietveld analysis and solid-state nuclear magnetic resonance) allowed to confirm crystal structure and silicon incorporation. Moreover, the obtained results suggested the presence of silicate and disilicate ions that partially substituted phosphate groups in crystallographic sites. Silicon substitution into the αTCP crystals resulted in a significant increase of *b* axis length and the β angle [99].

During the last few years αSiTCP has been also examined in vivo [101,102,103]. Cylindrical implants, ceramic blocks [102,103] or porous granules based on this material [101] have already been prepared and examined by conducting biological tests on rabbit and rat models. All the materials demonstrated good bioactivity and biocompatibility as well as mechanical properties [102]. What is more, the addition of Si promoted osteogenesis and again, retarded biodegradation of αTCP [101].

**Figure 6:**
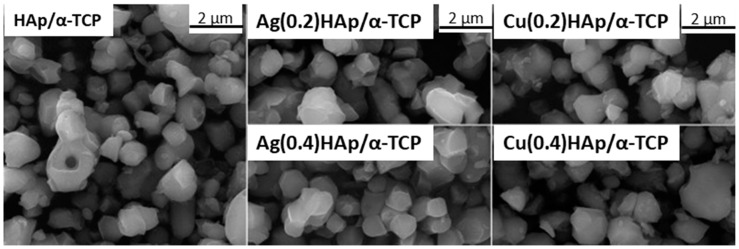
Field emission scanning electron microscopy (FESEM) micrographs of the powders calcified at 1200 °C (CuAg). Scale bar: 2 μm. Reprinted from [104] with permission from Elsevier.

Not only silicon has been used as a ionic modifier of αTCP. The study [104] considered applying a hydrothermal method to obtain hydroxyapatite (as a precursor of αTCP) modified with Cu^2+^ and Ag+. The as prepared HA powder was put under the temperature of 1200 °C for two hours in order to examine the influence of dopants on the creation of biphasic HA/αTCP powder. The aim of the study was also to investigate the antimicrobial activity of the prepared material. All of the annealed powders consisted mainly of spherical particles (see Figure 6). The doping of foreign ions had slightly negative influence on the creation of biphasic material. The antimicrobial activity of both modified HA and biphasic powder were evaluated against *Staphylococcus aureus*, *Escherichia coli*, *Pseudomonas aeruginosa*, and *Candida albicans*—the bacterial and yeast strains responsible for common human infections. 

Although the tests showed that more ions were released from the doped HA, the biphasic powder performed more homogenous and, in some cases, better antimicrobial activity than modified HA. The authors believe that this phenomenon can be explained by the adsorption of microorganisms on the surface of the HA/αTCP, which put them in close contact with antimicrobial agents, i.e., Ag^+^ and Cu^2+^, preventing further expansion [104].

In turn, Tong et al. [106] presented an unconventional approach, which examined the introduction of foreign ions into αTCP. Using a solid-state technique, they modified the CaP with Eu^3+^. The outcomes of the study suggested that, due to the self-reduction, europium could serve as a spectroscopic probe for the detection of αCa_3_(PO_4_)_3_ in the phase transition process.

#### 4.3.2. βTCP Substituted with Foreign Ions

Among non-apatitic CaPs, the most thoroughly investigated material considering ionic substitution is βTCP [108,109,110,111,112,113,114,115,116]. In turn, magnesium is definitely the most widely incorporated dopant into its crystal lattice [108,109,111,112,115,116,117,118,119,121,122,123,124,125]. The most ubiquitous method of obtaining Mg-substituted βTCP is annealing already precipitated CDHA powders modified with Mg^2+^. Analysing the behaviour of these materials during heat treatment, several main trends may be observed. Introducing Mg^2+^ lowers the thermal stability of HA and therefore facilitates CDHA’s transformation into βTCP. In study [118] the process occurred within the temperature range of 600 to 700 °C in case of Mg-substituted CDHA, whereas in the absence of the dopant, the phase transition took place at higher temperatures (700 to 800 °C). Introducing magnesium into βCa_3_(PO_4_)_3_ suppresses β to α phase transformation. Marchi et al. proved that the addition of at least 1.5 mol % is enough to retard the above process [119].

The maximum substitution of Mg^2+^ in Ca^2+^ sites has been also examined and varies from 14 to 15 mol % [115,116]. Mg ions occupy Ca(4) and Ca(5) sites in the crystal structure. Successful attempts were made to create interconnected micro and macroporous Mg-substituted βTCP scaffolds. 

**Figure 7:**
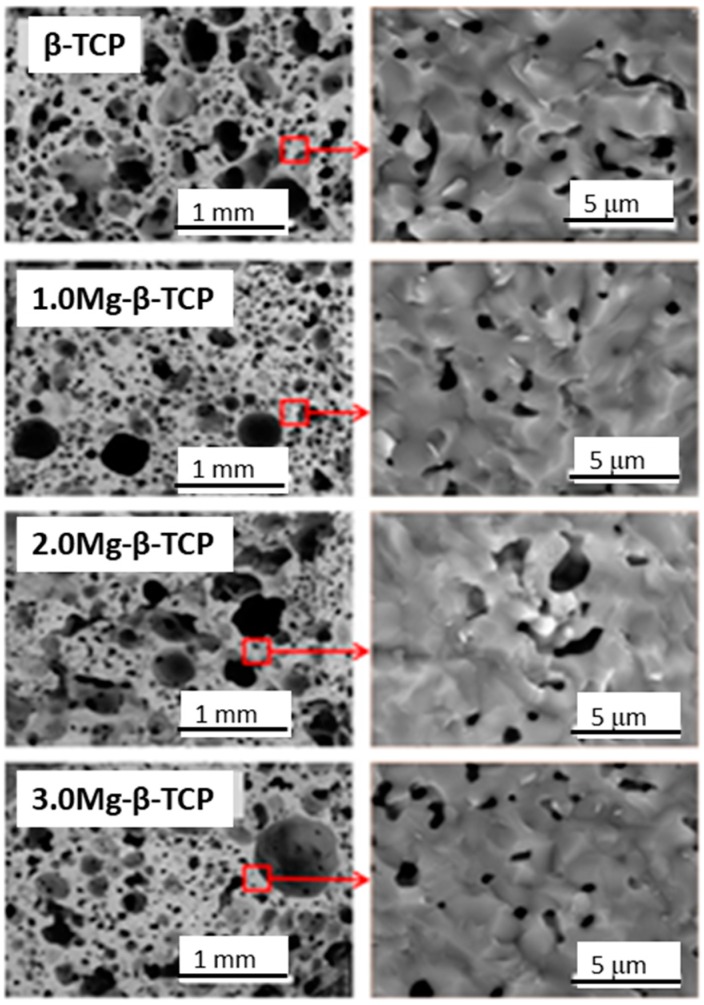
FESEM images Mg-containing micro-macroporous scaffolds fracture surfaces. Reprinted from [123] with permission from Elsevier.

In study [123], porous structures were obtained as an effect of the in situ foaming of the examined slurry. NH_4_HCO_3_ was used as a foaming factor. Subsequently, the viscous mass was sintered at 1150 °C for two hours. The field emission scanning electron microscopy (FESEM) images of these prepared materials are presented below (see Figure 7).

Another method to create βTCP—based porous scaffolds was applied in study [122]. Briefly, βMgTCP powder was made into slurry with distilled water and polyvinyl alcohol. Subsequently, polyurethane foams were soaked in the previously prepared slurry (see Figure 2) [122]. The excessive slurry was removed by squeezing. The soaked foams were dried and put under a heat treatment of above 1500 °C. The sintering temperature was higher than the β to αTCP transition temperature. The results of the study showed that the addition of magnesium was crucial to prevent the transformation during heat treatment. 

It is not only magnesium that is being introduced into the βTCP crystal lattice. In the literature, following examples of other ionic modifications can be found: sodium [127,143], potassium [128], silver [135], manganese [132], silicon [111,133], strontium [107,112,113,114], copper [136], cobalt [137], aluminium [146], iron [139,140,141], lantanium [131], and rare earth elements [145].

Divalent cobalt ions (Co^2+^) were introduced into the βTCP structure due to their potential induction of angiogenesis process [137]. The physicochemical and biological properties of the obtained materials containing 2 and 5 mol % of Co^2+^ were analysed. The results suggested that, similarly to magnesium, the addition of cobalt ions stabilizes the βTCP phase. The samples performed also low toxicity and stimulated significantly the synthesis of the vascular endothelial growth factor (VEGF).

In turn, studies conducted by Kannan et al. [126,127,128] investigated the influence of sodium, potassium and chlorine on CaP formation. Firstly, aqueous precipitation was applied to obtain CDHA modified with Na^+^, K^+^ or Cl^−^. NaNO_3_, KNO_3_ or NH_4_Cl were used as sources of dopants. The prepared powders were calcified at the temperature of 800 °C. Physicochemical studies confirmed successful ionic incorporation in each case. The resulting materials were biphasic mixtures consisting of HA and βTCP modified with the appropriate elements. The main effect of introducing monovalent ions into the HA crystal lattice was an increase in its thermal stability to the temperature of between 1200 and 1300 °C [127].

Recent works also focused on iron-doped βTCP [139,140,141]. Considering the fact that CPCs substituted with magnetic ions (i.e., Fe^3+^) exhibit ferromagnetic properties, they may be used as heat mediators in hyperthermia treatment of tumors, drug delivery systems or biomaterials for magnetic resonance imaging (MRI) [141]. The studies provided by Singh et al. [140] indicated that addition of the Fe^3+^ ions preserved the structural stability of βTCP. The limit of iron substitution was found to be 5.02 mol %. It was also shown that only high content of Fe^3+^ ions introduced into βTCP structure may produce pronounced hyperthermia effect. Detailed studies on ferric doping mechanism was presented in [141]. Hyperthermia effect was investigated on Fe^3+^/Ni^2+^ co-substituted βTCP [139]. High nickel content exhibited significant cytotoxicity. However, iron and nickel co-substitution displayed optimal hyperthermia effect.

The potential antibacterial activity of βTCP substituted with appropriate ions was also investigated. Gokcekaya et al. [135] and Rau et al. [136] provided data considering the activity of AgβTCP and CuβTCP, respectively. Small amounts of silver or copper ions were nontoxic for human cells and effectively lethal for bacteria. Moreover, in one of the studies, Matsumoto et al. [129] presented the effects of co-doping βTCP with monovalent and divalent antibacterial ions. They introduced Ag^+^ and Cu^2+^ or Zn^2+^ using a solid-state method. The Ag^+^ concentration was constant and maintained at the level of 9.09 mol %, while the amount of Cu^2+^ or Zn^2+^ varied from 0 to 15 mol %. The antibacterial activity of synthesized materials against *S. aureus* and *E. coli* was evaluated. Interestingly, co-doped powders exhibited better antibacterial effect than AgTCP and pure TCP. What is more, the release rates of the ions from CuAgTCP and ZnAgTCP were slower than in case of AgTCP and unsubstituted TCP. Hence, the results of the study strongly suggest that co-doped materials can be used over a long period of time, exhibiting a good antibacterial activity [129].

Not only antibacterial ions are being introduced into βTCP. Scientific research is also focused on loading porous, modified βTCP nanoparticles with drugs, which are commonly used to treat hard tissue infections [114,124]. An interesting method to obtain porous, doxycycline loaded material was applied in study [124]. Mesoporous MgβTCP nanospheres were obtained from amorphous Mg_2_P_2_O_7_ using EDTA ions as a nucleating factor. Subsequently, the prepared material was loaded with the antibiotic by immersion. The nanoparticles exhibited sustained drug release and easy cell reuptake [124].

A simultaneous introduction of more than one element into βTCP structure is also being investigated. In studies [142,143], magnesium ions were co-substituted together with strontium [142] or sodium ions [143]. All the structures were determined using detailed X-ray diffractometry and Rietveld analysis. The obtained results showed that Mg^2+^ ions play the crucial role in the significant reduction of the *a* and *c* axis lengths. It was also revealed that Sr^2+^ occupy Ca(1,2,3,4) sites whereas Mg^2+^ is located at the six fold coordinated Ca(5) site [142]. Thermal stability as well as the limit of substitution was examined. An interesting work was presented by Meenambal et al. [144]. A simultaneous substitution of Gd^3+^ and Dy^3+^ into the βTCP crystals was investigated. The substitution limit for co-substituted ions was determined as ca. 2.2 mol % and was sufficient to exhibit paramagnetic effects. Biological tests revealed negligible toxicity of the obtained materials. Therefore, this new material may find potential application as multifunctional bioprobe or a contrast agent in MRI and CT (Computer Tomography) [144]. The simultaneous introduction of more than two ions into the βTCP crystal lattice has been also conducted. Bose et al. [108] applied a solid-state technique to obtain βCa_3_(PO_4_)_3_ modified with magnesium, strontium, and silicon ions at the same time. SrO, MgO, and SiO_2_ were used as dopants sources.

## 5. Conclusions

The ideal bioceramics for bone grafting must combine several biological properties such as bioactivity, biocompatibility, osteoconductivity, and non-immunogenicity. Moreover, these materials should possess mechanical resistance together with suitable resorbability, which allows for bone remodelling and new bone formation.

Different strategies can be taken to improve the biological properties of CaP bioceramics. One of them is to enrich the materials with particular ions, which, even in small amounts, have significant impact on the osseous cell activity, mechanical and thermal properties and solubility.

This review provides evidence for the growing interest in substituted, non-apatitic CaPs. Until now, this research has focused on these ions, which are favourable considering the biocompatibility, bioactivity, and osteointegration of synthesized materials. There has not yet been sufficient investigation in the field of ionic substitution providing biological properties i.e., antimicrobial or anticarcinogenic. We think that co-substitution in non-apatitic CaPs may represent a considerable field of scientific research, too.

## Figures and Tables

**Figure 8:**
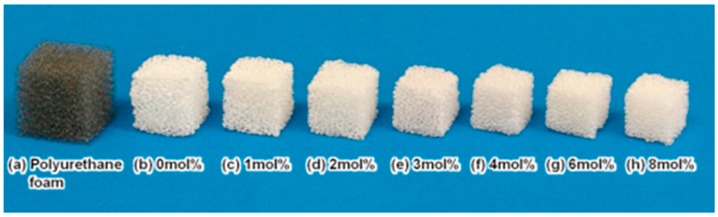
Typical macroscopic structure of: (**a**) the polyurethane foam template and (**b**–**h**) TCP foams prepared with calcium phosphate slurries containing different amounts of MgO. Reprinted from [122] with permission from Elsevier.
